# The effectiveness and cost-effectiveness of a participative community singing programme as a health promotion initiative for older people: protocol for a randomised controlled trial

**DOI:** 10.1186/1471-2458-11-142

**Published:** 2011-02-28

**Authors:** Ann Skingley, Stephen M Clift, Simon P Coulton, John Rodriguez

**Affiliations:** 1 Sidney De Haan Research Centre for Arts & Health, Canterbury Christ Church University, University Centre Folkestone, Folkestone, UK; 2 Centre for Health Service Studies, University of Kent, Canterbury, Kent, UK; 3 Eastern and Coastal Kent Primary Care Trust, Dover, Kent, UK

## Abstract

**Background:**

The growth in numbers of older people represents a considerable cost to health and social care services in the United Kingdom. There is an acknowledged need to address issues of social exclusion and both the physical and mental health of this age group. In recent years there has been much interest in the potential contribution of the arts to the health of communities and individuals. There is some evidence that participative singing may be of benefit to older people, however studies to date are limited in number and have lacked rigour. There is therefore a need to build on this knowledge base to provide more quantifiable evidence of both effectiveness and cost effectiveness of singing as a health intervention for this population group.

**Methods:**

The proposed study is a pragmatic randomised controlled trial with two parallel arms. The primary hypothesis is that singing groups for older people improve both physical and mental aspects of quality of life when compared to usual activities. Potential participants will be volunteers over 60 years living in the community and recruited through publicity. Eligible and consenting participants will be randomized to either a singing group or a control group. Singing groups will take part in a twelve week planned programme of singing and control groups will continue with usual activities. The primary outcome measure will be the York SF-12, a health related quality of life measure which will be administered at baseline, three and six months after baseline. The study will evaluate both effectiveness and cost-effectiveness.

**Discussion:**

This study proposes to add to the existing body of evidence on the value of singing for older people by using a rigorous methodological design, which includes a power calculation, a standardised intervention and assessment of cost-effectiveness. It should be regarded as a stage in a progressive programme of studies in this area. If group singing is found to be effective and cost-effective it may offer an alternative means to maintaining the health of people in later life.

**Trial Registration:**

ISRCTN62404401

## Background

The growth of the older population in the UK is substantial and the social care and health needs of older people represent a considerable cost to health and social care services [1]. There is clear scientific evidence of the importance for 'successful aging' of maintaining an active lifestyle-physically, mentally and socially [2] and considerable attention has been given to the need to promote the health of older people, to help them maintain active and independent lives in the community and delay the need for residential care [3]. There is also a need to address mental health problems among the elderly and wider issues associated with social isolation and exclusion.

It is widely understood that the NHS cannot alone address the diverse needs of older people, but has to work in partnership with other statutory bodies and voluntary agencies in the development of services and models of intervention to assist people in living independently and maintaining health and wellbeing [4-7].

In recent years there has been growing interest in the potential value of the arts in addressing significant social issues [8,9]. The social utility of the arts has been promoted by the national Arts Councils, and both DCMS and DH have supported research to explore the contribution of the arts in the field of mental health[10]. The Department of Health established a Review of Arts and Health Working Group which reported in 2007. This led to the Department of Health and Arts Council England publishing A Prospectus for Arts and Health (2007)[11], which provides an overview of the current Arts and Health field in the UK, and offers recommendations for future developments. Coinciding with publication of the Prospectus, Arts Council England published its own strategic framework on Arts, Health and Wellbeing [12].Under the Labour Government the Home Office recognised the value of the arts in work with offenders, and the Treasury funded several projects on arts and community health under the Invest to Save Budget framework (see for example Manchester Metropolitan 'Invest to Save Arts in Health Project' http://www.miriad.mmu.ac.uk/investtosave/.

There is growing recognition of the value of arts activities in improving the lives of older people [13-16], and particularly the value of live music and musical participation for older people [17], including those affected by Alzheimer's and other forms of dementia [18,19]. These studies are useful in highlighting some of the standardized measures which may be employed in studies of arts and health. They do, however, address very disparate interventions within a single study, making cause-effect conclusions problematical. Further, many studies use subjects as their own controls, rather than incorporating a control or comparison group, which would further enhance scientific rigour.

We have recently completed a systematic review of all available non-clinical research studies concerned with the possible benefits of active engagement in singing. A systematic search of the published literature identified 56 reports giving attention to singing and its benefits. Of these reports, 18 were excluded from consideration as being of limited interest. The remaining 36 sources are extremely diverse with respect to the research problem addressed, the participants involved, research design and methods of data gathering and analysis. These papers have been carefully scrutinised but limited synthesis is possible.

Only two studies out of these 36 attempted to assess the wellbeing/health benefits of participation in group singing using standardised measures of health [20,21]. Both studies were concerned with older people. In each study, a positivistic research model was adopted in which pre and post assessments were made of participants in intervention and control groups, with statistical comparison of means between the groups.

Houston et al [20] (UK) assessed the impact of four hour-long sessions of singing over four weeks with 31 residents in three care homes using GHQ-28 and HADS. A non-intervention group of 30 residents in three further care homes were also assessed (mean age 84 years). The authors claim that the intervention group showed significant improvements in measured anxiety and depression over the four weeks compared with the non-intervention group.

Cohen et al. [21] (USA) assessed the impact of weekly participation in a community choir on 90 people aged 65 and older. The study ran over a period of two years, and a range of measures of physical health, health service utilisation, mental health and social activity were employed at pre-test and follow up after one and then two years. A matched non-intervention comparison group (N = 76) were also assessed. The authors claim to find a range of positive effects from participation in the singing group, including higher rating of physical health, fewer doctor visits, fewer falls and better mental health.

Both studies have limitations and both methodological and analytical weaknesses, which raise doubts about the validity of their conclusions. Neither study justified sample sizes in terms of study power. In neither study is there evidence of randomization to intervention or control group, or any formal assessment of cost effectiveness of the intervention.

A central focus of the current proposal is the evaluation of an innovative initiative - the Silver Song Club Project - which provides opportunities for older people to come together on a regular basis to make music and sing, with the support of professional musicians and volunteers drawn from established choral societies and singing groups. Currently, over 40 Silver Song Clubs are in operation across the South East of England, managed by a third sector organization, Sing For Your Life Ltd. (SFYL).

A qualitative and process-oriented formative evaluation has been completed [22], providing information on how the project is run, the experiences of facilitators, volunteers, participants and carers and perceived benefits gained. Data were collected on six of the clubs through observation of sessions, focus groups with volunteers and interviews with club facilitators and participants, venue managers and SFYL directors and facilitators. Older participants have reported positive health benefits across psychological, cognitive, social and physiological domains, supporting previous research findings. There is now a need to develop a more controlled and objective assessment of the benefits for older people of participation in Silver Song Club activities.

### Aims of the study

1. To assess the effectiveness for older people of active engagement in community music activities on measures of physical and mental health.

2. To evaluate the cost-effectiveness for older people of active engagement in community singing.

## Methods

The study is a pragmatic randomised controlled trial with two parallel arms. The study has been granted ethical approval by Surrey Research Ethics Committee REC ref: 10/H1109/5 and complies with the Helsinki Declaration.

### Hypotheses

#### Primary hypothesis

Stated as null hypothesis

Singing groups for older people are no better than usual activities in increasing health-related quality of life in older people measured six months after randomisation using the York SF-12.

#### Secondary hypotheses

Stated as null hypotheses

1. Singing groups for older people are no more cost-effective than usual activities

2. Singing groups for older people are no better at reducing anxiety and depression when compared with usual activities at six months after randomisation measured using the Hospital Anxiety and Depression Scale (HADS).

### Participants

#### Inclusion criteria

The study will, as far as possible, reflect the inclusive nature of existing Silver Song Clubs. Participants will be adults over 60 years of age, able to speak English and able both to provide informed consent and to complete questionnaires.

#### Exclusion criteria

Individuals unable to provide informed consent or complete questionnaires in English.

#### Recruitment

Researchers, in conjunction with the third sector partner organization providing the intervention, will seek suitable venues across East Kent for song clubs to take place. Adverts will be placed in the venues and local newspapers and leaflets will be delivered to homes within the vicinity of the venues. These will specify the locality of the five venues and request that interested individuals should specify a locality to which they will be attached should they decide to take part. Adverts will also state that participants will be randomly allocated into either the singing group or the 'usual activities' group within that area. Local radio publicity will also be sought. Two 'taster sessions' will be held in each venue which will include the provision of information, an invitation for questions and an opportunity for individuals to sample the nature of the proposed intervention programme. A dedicated phone line and email address will be set up to deal with responses and queries and a proforma will be devised to collate details of those volunteering to participate. Personal details will be held in a locked filing cabinet within the research centre.

#### Randomisation and consent

All individuals indicating an interest in taking part will be sent a baseline questionnaire. They will also be sent an information sheet outlining the purpose of the study and what to expect if taking part, plus a consent form to be returned with the questionnaire.

All returned, consented questionnaires will be randomly assigned to either intervention or control arm within one of five geographical areas. Randomisation will be conducted using the secure remote randomisation service based at the University of Kent. Because of the known preponderance of women in singing groups, stratification by gender will be used. Individuals who would attend together if allocated to a singing group (e.g. couples or friends providing transport) will be randomised in pairs on request.

### Intervention

The Silver Song Club model is a well established format for participative singing for older people and this will be replicated in a standardised form. Trained and experienced facilitators under the guidance of SFYL will meet to compile a twelve week 90 minute programme comprising songs from different eras and a variety of genres. This will be followed by a series of 'unification' meetings, to ensure that all facilitators are aware of how to access the material and deliver it in the same way (e.g. accompaniment, key, acquiring copyright etc). The programme will be developmental, progressing from singing melody lines to harmonising, layering and singing in rounds. Chime bars will also be introduced where appropriate and there will be an opportunity for participants to request items. All clubs will deliver the same programme concurrently, and consistency will be monitored by a programme manager who will make unannounced visits to each club 5-6 times during the intervention. A song book will be especially produced for the trial and a register of attendees will be maintained.

### Control

Individuals in the control group will continue with their normal activities. Should they decide to join a singing group outside the research they will not be excluded but data will be collected on these activities.

### Sample size

A power calculation was carried out for the primary outcome (health related quality of life measured on the York version of the SF-12). There are no previous randomised controlled trials evaluating the impact of singing groups on quality of life in this population. Using data from quasi-experimental and observational studies we hypothesise that a minimally important difference in health related quality of life between the intervention group and control groups is in the order of 5 points on the York SF-12, equivalent to a medium effect size difference of 0.5. To detect this difference using a two-tailed test, alpha of 0.05 and power at 80% requires 63 participants in each of the two arms, a total of 126. We anticipate 5 singing groups and 5 controls and need to take account of any clustering effect in calculating sample size. We have used a conservative estimate of intra-class correlation coefficient of 0.02, similar to general practice populations and a cluster size of 12. This inflates the required sample size by a factor of 1.2, 77 in each group, a total of 154. Previous research would suggest the loss to follow up at 6 months for this population would be in the order of 20% and this further inflates the sample size to 92 in each group, a total of 184. We do not anticipate that all those enquiring would be willing to take part in the evaluation but we do have evidence that the consent rate would be in the order of 70%. This would mean we would require a total of 240 potential participants, 24 in each singing group and control group over the course of the study.

### Outcomes

The study will use standardised instruments with demonstrated validity, reliability and responsiveness. In addition, data will be collected on health and social care service utilisation and activities (including musical and singing activities) engaged in over a period six months prior, and six months after randomisation. Outcomes will be measured using a postal questionnaire.

#### Primary outcome

The primary outcome measure will be self-assessed health related quality of life assessed using the York SF-12 [23]. This is a frequently used measure that has been validated for use with older people and for which population norms exist. The twelve multiple choice questions cover both physical and mental domains of health. Most questions ask about health over the past week. Measures will be taken at baseline (prior to randomisation), 3 and 6 months after randomisation. The instrument allows the generation of an overall health-related quality of life score and additional two component scores addressing physical and mental health aspects of health-related quality of life.

#### Secondary outcomes

Health utility will be measured using the EQ-5D [24]. This is a short, 3-level, 5-dimensional instrument allows the generation of Quality Adjusted Life Years. It is routinely used in the economic evaluation of health care and recommended for cost-effectiveness analyses.

Anxiety and depression will be measured using the Hospital Anxiety and Depression Scale (HADS) [25]. This has validity for use in community as well as hospital settings and has been used in previous studies evaluating arts and music interventions.

Health and social care service utilisation will be measured using a specially designed questionnaire used in a number of evaluations involving older populations.

All secondary outcomes will be measured at baseline, and then 3 and 6 months after randomisation.

In addition we will collect process data consisting of individual attendance at singing groups for participants and information on who delivered singing group sessions.

### Data analysis

#### Effectiveness analysis

Data analysis will be conducted blind to the allocation of participants. The primary analysis will be intention to treat, where participants are analysed as part of their randomised group irrespective of their attendance. This provides the most pragmatic estimate of effectiveness. The primary outcome measure, the SF-12, will be analysed at 6 months using analysis of covariance to adjust for baseline differences between the groups. Due to the clustered nature of the study in which participants are nested within singing groups multi-level modelling will be undertaken to adjust for any cluster effects. Results will be presented as means and 95% confidence intervals. Secondary outcomes will be analysed in a similar manner. We will also explore the potential efficacy of the intervention by conducting a per protocol analysis in which only those participants who attended at least 50% of sessions are included. Other secondary analysis will include a regression analysis to explore both potential predictors of outcome and any baseline x treatment interactions.

#### Cost-effectiveness analysis

The incremental cost-effectiveness of singing groups compared with usual group activities will be assessed from both a health and personal social services perspective using NICE guidelines [26] and a wider public sector resource perspective [27]. The costs of setting up singing groups will be assessed using the actual resource costs for these activities based upon local service costs including costs associated with premises and managerial overheads. The costs of providing singing groups will be based on information gathered regarding participant contacts (all intervention contacts the participants receive, including the time and resources used to deliver the song groups which the participants attended) throughout the study. The participants' use of health, social care or welfare services at baseline, 3 and 6 months will be calculated from the service use questionnaire. Units of resources used will be combined with national sources of unit costs [28,29]. Because the resource use occurs within a 12 month period no discounting will be applied. The EQ-5D will be used with population values and the quality adjusted life year (QALY) change calculated using the area under the curve method. Bootstrapping will be conducted to explore the sensitivity and stability of derived estimates and incremental cost-effectiveness ratios and cost-acceptability curves presented.

## Discussion

The aim of this study is to evaluate the effect of group singing on the health related quality of life in older people. The existing body of evidence suggests that singing may be of benefit to this population group, but leaves many open questions. The two most relevant published controlled studies have significant methodological limitations, which call into question the credibility of findings.

This study proposes to advance knowledge through a number of mechanisms, including: the use of a scientifically rigorous methodological design, use of a power calculation to estimate sample size, a standardized intervention which can be replicated, the assessment of cost-effectiveness of the intervention and single-blind analysis.

The proposed research should be regarded as a step in a progressive programme of studies investigating the value of group singing for older people. Taking the MRC framework for the evaluation of complex interventions [30] as a model, this protocol represents the second (evaluation) stage, building on the feasibility study (our formative evaluation - [22]). Further refinements may be undertaken in the future, building on this study and dependent upon greater resources. This may include: offering an alternative activity for the control group, a longer term follow-up and a greater geographical spread.

If group singing proves to be both effective and cost-effective, it may offer an alternative to more expensive, labour-intensive and less enjoyable means to maintaining the health of people in later life. This would be beneficial to the NHS and social care organizations as well as to individuals, groups and communities.

## Conclusions

This study is, we believe, the first to investigate the health benefits of singing for older people based on a stringent randomised controlled trial design which includes a cost-effectiveness evaluation. Its intervention, based on an existing model of participatory singing for older people, maximizes its potential for acceptability as a health promotion initiative for this age group should the outcome prove positive.

## Competing interests

The authors declare that they have no competing interests.

## Authors' contributions

AS drafted the manuscript and is coordinating the study; SMC conceived of the study, participated in the design and is chief investigator; SPC revised the manuscript and advised on the design and data analysis; JR is principal investigator and NHS lead. All authors read and approved the final manuscript.

## Pre-publication history

The pre-publication history for this paper can be accessed here:

http://www.biomedcentral.com/1471-2458/11/142/prepub
